# Segregated peripheries of Gran Valparaíso, Chile: new walls for social-urban integration in the Chilean city

**DOI:** 10.3389/fsoc.2026.1735352

**Published:** 2026-04-29

**Authors:** César Cáceres-Seguel, Juan Correa-Parra

**Affiliations:** 1 Centro Regional de Inclusión e Innovación Social, Universidad Viña del Mar, Viña del Mar, Chile; 2 Facultad de Arquitectura, Universidad Gabriela Mistral, Santiago, Chile

**Keywords:** peripheries, residential segregation, social housing, urban policies, Valparaíso

## Abstract

The Latin American city is structured by structural social inequality, expressed in an inequitable distribution of people and urban goods across the city. Therefore, analyzing the degree of residential segregation associated with the expansion of these cities is fundamental for urban policy design. Using spatial statistical techniques, we analyze the level of residential segregation existing in the urban expansion areas between 1992 and 2017 in the Valparaíso Metropolitan Area (MAV), Chile. The results show that suburban expansion zones develop with higher levels of residential segregation than the rest of the city. Moreover, social housing subsidies aimed at the development of projects with social mixing are located in highly segregated areas. The pattern of highly socially segregated suburban areas challenges Chilean urban policy and its objective of social integration.

## Introduction

Historically, cities have been conceived as places where social needs are resolved. This explains why by 2050, 75% of the world's population will live in cities. This notion of the city as an instrument of social change explains why the UN explicitly urges governments to strengthen urbanization processes (Habitat World Map UN, 2019, p. 219). Despite this positive view of urbanization, the high levels of poverty, inequality, and lack of basic urban infrastructure present in Latin American cities call for a critical look at this assertion. In Latin America, 81% of the population lives in cities; therefore, challenges in reducing social inequality will largely be addressed in urban areas. In this discussion, the expansion areas of Latin American cities are strategic, as diverse projects and communities converge there: luxury condominiums, social housing, and informal settlements, not necessarily integrated into a metropolitan plan that guides the city functionally and socially. The peripheries of Latin American metropolises are often identified with population overcrowding, environmental degradation, and the presence of high levels of social homogeneity (Rasse Figueroa et al., 2021).

Chile is notable because almost ninety percent of its population lives in cities. An estimated 60% of its housing deficit is in its three main metropolitan areas. This urbanization happens in a country with high social inequality. The wage gap between minimum and managerial salaries is 102 times, much higher than the 32-fold OECD gap. The pursuit of equity has led to urban policy that seeks to reverse segregation and ensure access to public urban goods [Ministerio de Vivienda y Urbanismo (MINVU), 2014]. Since the late 20th century, states have made housing a pillar of social policy. Despite advances in housing coverage, design quality, and neighborhood facilities, debates continue. The main challenge is improving the location of social housing and reducing its concentration on city outskirts (Fuster-Farfán et al., 2023; Cáceres, 2017).

The urban expansion of the Valparaíso Metropolitan Area between 1992 and 2017 is analyzed in relation to the existing degree of residential integration or segregation. It is the third most important conurbation in Chile, after the urban areas of Greater Santiago (6,622,000 inhabitants) and Greater Concepción (1,040,000 inhabitants). It represents an interesting case, as it has undergone an intensive urbanization process. It is estimated that 80,000 housing units were built between 1992 and 2017 in the Valparaíso Metropolitan Area. Studies on the metropolitan area of Valparaíso have focused on sociodemographic characterization (Valdebenito et al., 2020); metropolitan growth trends (Fuentes and y Pezoa, 2017), and access to urban goods (Cáceres and y Ahumada, 2020). However, we do not yet understand the degree of residential segregation present between different areas that comprise the Metropolitan Area of Valparaíso. The questions guiding this study are: Is the suburban expansion of the MAV a territory built under high or low residential segregation? Do social housing programs focused on social integration tend to be in areas with a social mix? The objective of this research is to analyze the levels of residential segregation in the Valparaíso Metro in the Latin American city. Areas of suburban expansion are associated with issues of overcrowding, housing obsolescence, environmental degradation, and high degrees of social homogeneity (Rasse Figueroa et al., 2021).

Segregation operates through “vicious circles of segregation,” in which the place of residence determines access to the educational system, insertion into the labor market, and future income, which in turn affects access to the housing market—thus feeding back into educational and occupational opportunities. In this way, residential segregation is reinforced intergenerationally across time and space (Tammaru et al., 2021). Residential segregation in Chilean cities does not necessarily respond to racial or ethnic logics but rather to socio-educational or socioeconomic dimensions (Garreton et al., 2020), closely linked to housing markets and urban policies (Correa-Parra et al., 2023; Sabatini and Brain, 2008). Initially, it was understood that higher levels of social inequality would lead to higher levels of socio-spatial segregation in the city (the dual city notion of Sassen, 1991). However, studies such as those by Fujita (2012) in European and Asian cities show that the relationship between social inequality and segregation is not linear. The aforementioned author highlights that European or Asian cities with high inequality do not necessarily imply high segregation in their cities. A global perspective supports this relationship; studies across 24 large cities worldwide show that rising income inequality leads to rising socio-economic segregation almost everywhere. Differences in urban geography manifest as high-income workers often concentrating in city centers, attractive coastal areas, or gated communities, while low-income populations move toward urban peripheries or suburbanize. This dynamic results in increasingly segmented urban geographies where economic segregation is pronounced and changes rapidly, challenging social sustainability by making cities less inclusive (Van Ham et al., 2021; Tammaru et al., 2021).

*t* is important to study residential segregation not just as an outcome of economic differences, but also due to factors such as nationality or Indigenous status (Rojo-Mendoza and Alvarado Peterson, 2023; Ruiz-Tagle and López, 2014). This is especially relevant, as Chile's migrant population is rapidly increasing. In Chile, 12% of people declare Indigenous origin. It has been argued that measuring residential segregation only by income or access to goods can be misleading, as vulnerable groups sometimes gain goods through debt (Ruiz-Tagle and López, 2014). These trends create segregation among the most vulnerable in social housing peripheries (Rasse Figueroa et al., 2021; Cáceres, 2017). At the same time, elites self-segregate in gated communities or satellite cities (Sabatini et al., 2017). These inequality patterns are reflected in the uneven spread of services and amenities across the city.

Why is it relevant to analyze residential segregation in relation to the emergence of new peripheries? Several authors emphasize the need to study residential segregation in relation to the emergence of new peripheries within the city. Fernández-de-Córdova et al. (2021) highlight that processes of urban expansion reconfigure urban segregation by generating peripheral territories that are more socially homogeneous than the consolidated city. Rasse Figueroa et al. (2021) argue that the contemporary city produces new peripheries in expansion areas, adding to those already existing in the compact city. These areas are characterized by their distance from urban centers, physical distance between social groups, and the residential trajectories of their inhabitants, all of which pose specific characteristics and challenges. In this regard, the study shows that in these peripheral areas, the AMV intensifies social segregation compared to the compact city.

## Methods

A quantitative approach was used through an exploratory perspective that employs various spatial analysis tools to analyze the levels of residential segregation in the suburbs of the Valparaíso Metropolitan Area (VMA). Residential segregation levels were estimated based on the spatial distribution of socioeconomic groups defined by the Territorial Socio-Material Index (ISMT) methodology developed by the Observatorio de Ciudades UC (OCUC) (2023). This methodology is based on microdata from the national housing and population census conducted by the Instituto Nacional de Estadísticas (2017) and allows for estimating the socioeconomic status of a household based on data from four dimensions of analysis, corresponding to the level of education of the head of household, the material quality of the dwelling, the level of overcrowding, and the existence of additional occupants in the dwelling. These values are standardized using principal component analysis, and a score is determined on a household scale, ranging from 0 to 1, which represents the level of socio-material vulnerability of each household.

Subsequently, residential segregation levels were defined for the study area, for each of the census areas, using the information theory index (Hi) developed by Theil and Finizza (1971). This index is based on the development of an indicator that compares the diversity (heterogeneity, Hi) of local areas (in this case, census areas) with respect to the total diversity (the urban area of the AMV) that contains those local areas. In the case of a specific area (i), the index measures how the entropy of that area (Ei) is reduced in relation to the entropy of the region (E). Whereas the entropy of the region (E) is the weighted average of all the Hi values of the local areas that comprise it. In this case, T is the total population of the region, and yti is the population counted in area i. H represents the relative reduction in the average entropy of the components below the maximum entropy (E) recorded in that region Theil and Finizza, (1971).

The final result is an index ranging from 0 to 1, where values closer to 1 indicate that there is no diversity in the local area (census tract) analyzed, while values closer to 0 indicate that these local areas are as diverse as the region as a whole. In terms of segregation, values close to 1 are interpreted as higher levels of residential segregation, while values closer to 0 imply low levels of segregation. Once the levels of residential segregation and the socioeconomic groups involved were defined, households were counted according to the level of residential segregation in the census area (low, medium, high, in relation to the Theil index) and the predominant socioeconomic group in that census area (low, medium, high, in relation to the Territorial Socio-Material Index).

Finally, residential segregation levels were analyzed in relation to existing residential typologies in the outskirts of the MAV. First, to define the peripheral areas of the study area, all census areas that were not part of the MAV urban sprawl in 1992 were identified, based on the consolidated urban areas (AUC) defined by the Ministerio de Vivienda y Urbanismo (MINVU) (2020). These census areas were treated as the peripheral areas resulting from urban growth between 1992 and 2017.

## Results

**Figure 1:**
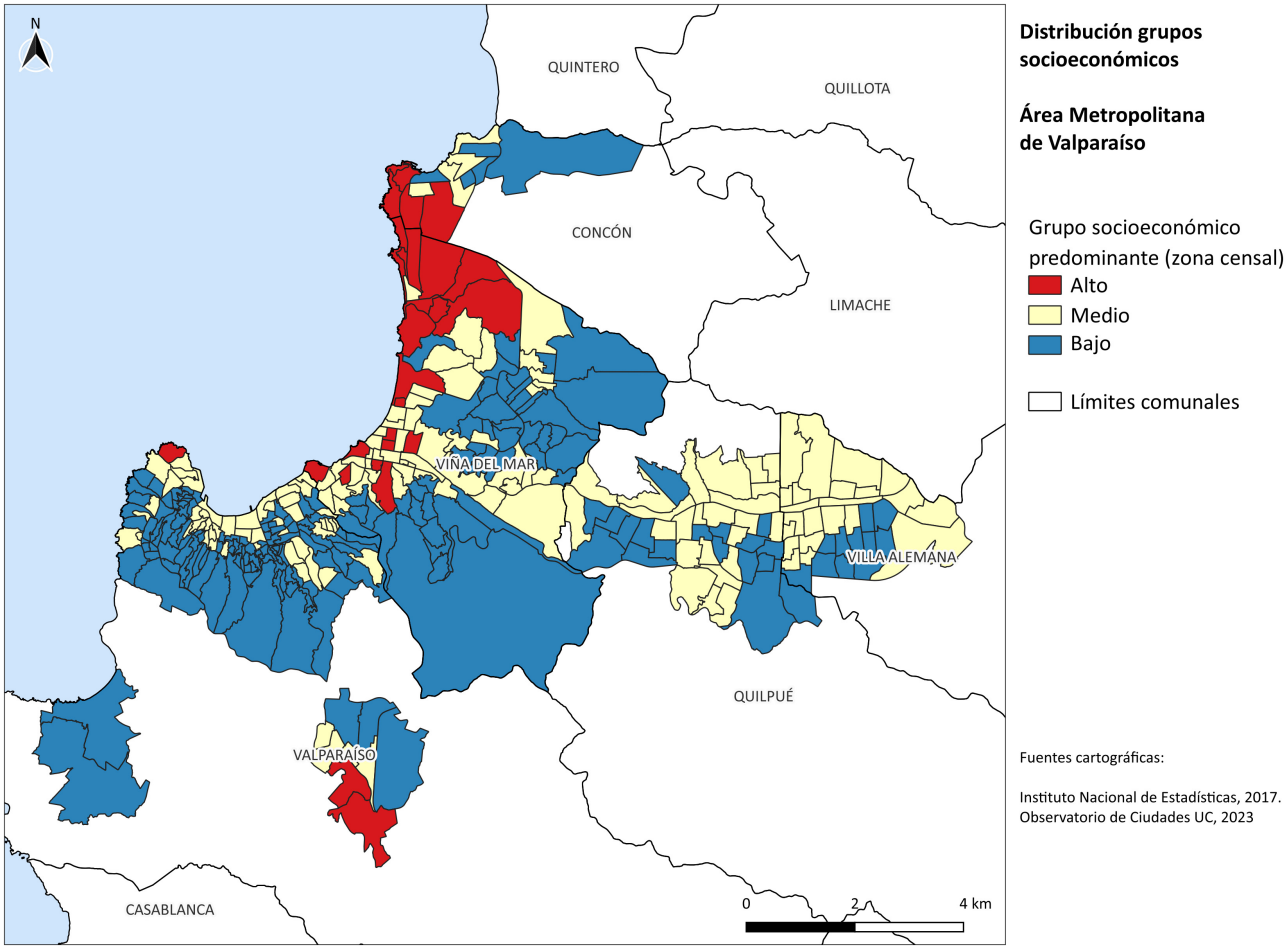
Predominant socioeconomic level by census tract in the VMA for the year 2017. Source: Own elaboration based on Observatorio de Ciudades UC (OCUC) (2023).

According to data from the Territorial Socio-Material Index of Observatorio de Ciudades UC (OCUC) (2023), the Valparaíso Metropolitan Area (VMA) is home to 303,013 households (6.4% upper-income group—ABC1; 41.5% middle income group—C2/C3; and 42.1% lower income inhabitans D/E). This is a metropolis predominantly composed of vulnerable groups living in the upper areas of Viña del Mar and Valparaíso, as well as in the southern peripheries of Quilpué and Villa Alemana (see Figure 1). At the other end of the social spectrum, high-income groups reside near the coastal areas, in the central polygons of Viña del Mar, and in gated communities in the Curauma area (southern periphery of Valparaíso).

**Figure 2:**
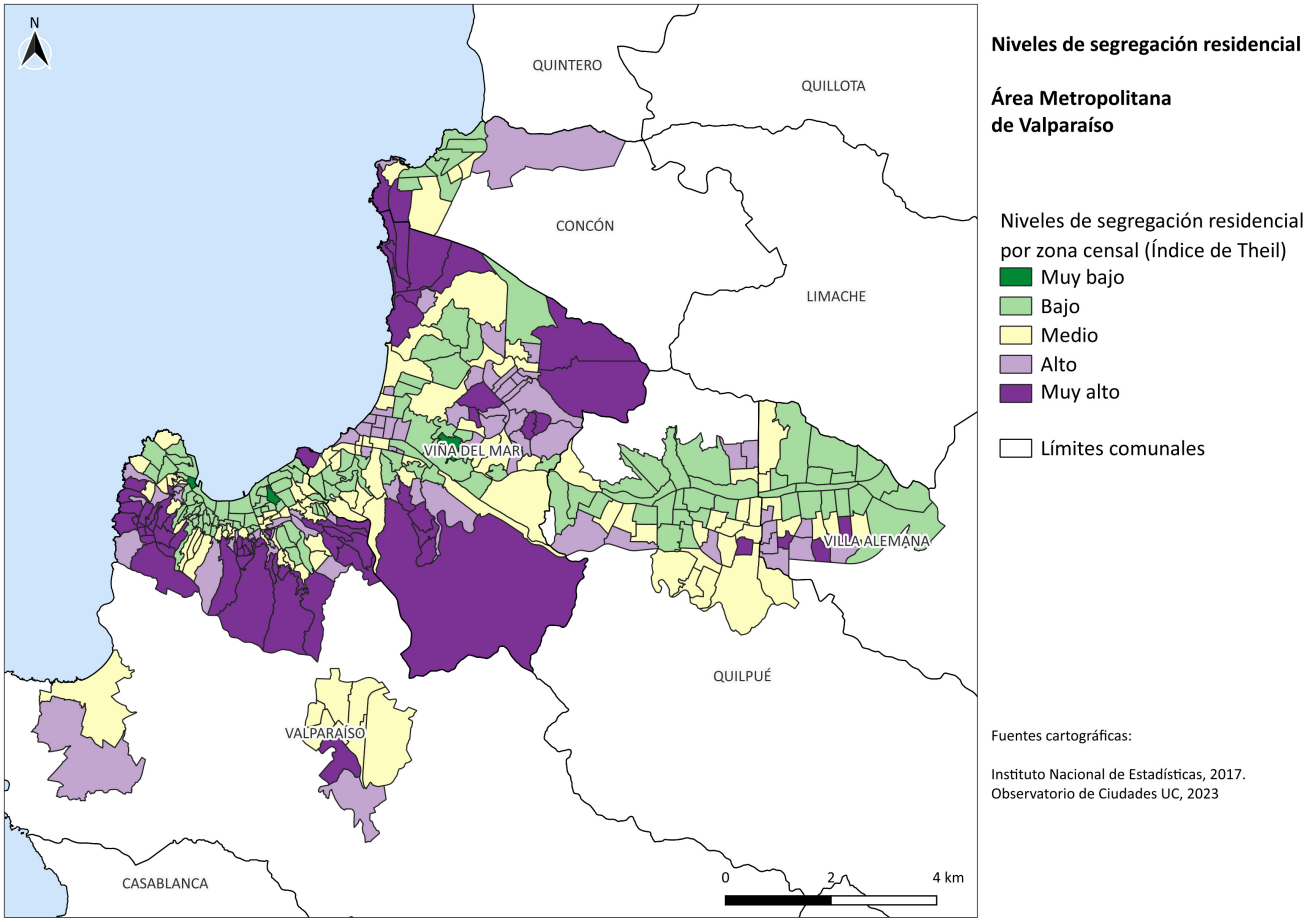
Residential segregation levels by census tract in the VMA for the year 2017. Source: Own elaboration based on Observatorio de Ciudades UC (OCUC) (2023).

Significant differences are observed within each municipality. When analyzing the entire urban area of the Valparaíso Metropolitan Area, we find that 16.7% of residents live in highly segregated zones; 48.2% reside in census areas with medium residential segregation; and 35.1% in areas with low segregation. Only Viña del Mar displays a central area with high residential segregation, characterized by the concentration of upper socioeconomic strata. In contrast, when analyzing the level of residential segregation specifically in the residential expansion areas of the MAV between 1992 and 2017 (see Figure 2), the proportion of households living under high residential segregation increases substantially to 26.4%. Meanwhile, households living in areas of low segregation within the suburban zones of the MAV account for only 18.7%. In other words, the residential areas developed since the 1990s, a decade marked by the consolidation of neoliberal urban policies, are more segregated than the MAV as a whole.

**Figure 3:**
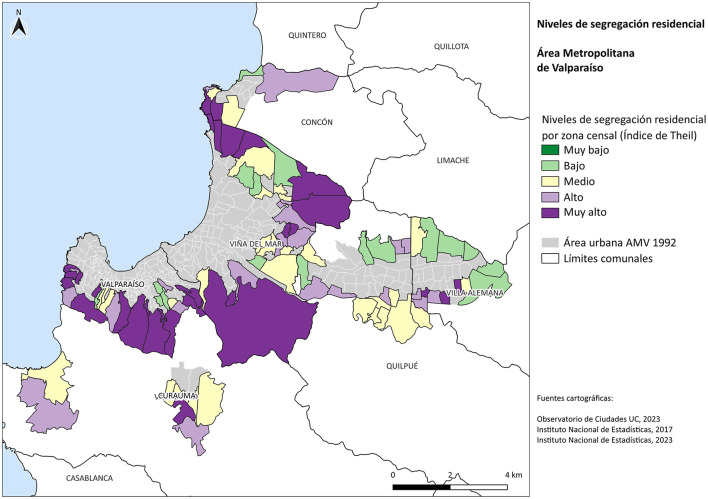
Residential peripheries of the valparaíso metropolitan area 1992–2017. Source: Own elaboration based on Observatorio de Ciudades UC (OCUC) (2023).

In the suburban expansion areas of the MAV, 54.9% of high-income groups live under conditions of high segregation. When examining this group across the entire MAV, this figure decreases to 41.1%, indicating that affluent residents are more socially mixed in the inner city. In the case of vulnerable groups, 26% live in highly segregated areas across the MAV as a whole, while in suburban expansion zones this figure rises to 42%. In the peripheries, the most vulnerable individuals experience a reinforcement of their spatial exclusion. For middle-income groups, a similar pattern is observed: in inner urban areas, only 3% live in high-segregation areas, whereas in suburban expansion areas, this percentage increases to 8% (see Figure 3). In summary, the residential expansion areas of the MAV between 1992 and 2017 are configured as spaces that operate as a social centrifuge, further distancing social groups from one another.

### Analysis by commune

**Figure 4:**
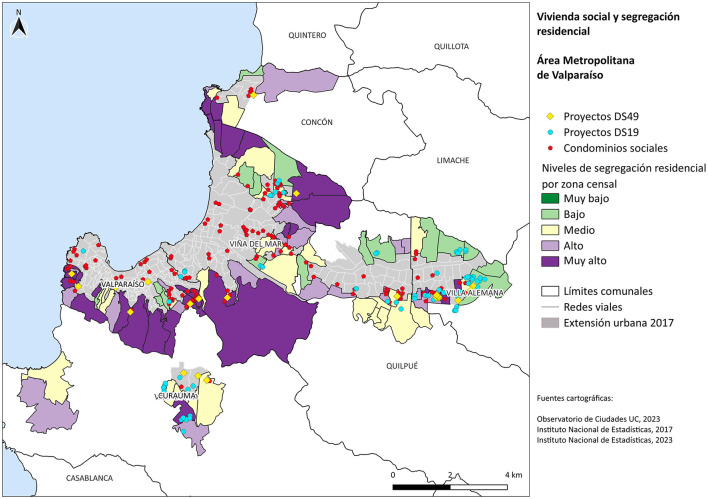
Social housing complexes in relation to residential segregation levels by census tract in the vma peripheries for the year 2017. Source: Own elaboration based on Observatorio de Ciudades UC (OCUC) (2023).

In the city of Valparaíso, the upper areas of the hills (above Avenida Alemania) are inhabited by highly segregated vulnerable groups, with the exception of specific cases—such as the San Juan de Dios, Yungay, and Bellavista hills—which exhibit a greater social mix. This is related both to the historical concentration of low-income populations in informal settlements—on the highest parts of the hills and in the ravines Pino and Ojeda, (2013)—and to the location of social housing complexes over the past 20 years (as illustrated in Figure 4). In the private satellite city of Curauma (see Figure 4), a highly segregated area (middle–upper socioeconomic group) can be observed, explained by private developments that have created a privatized city adjacent to the traditional town of Placilla (see Figure 2). In this case, we see the same phenomenon described by Fuster-Farfán et al. (2023), who points to the development of areas characterized by a strong center-periphery relationship (due to this suburb's dependence on access to employment opportunities) but also due to its relationship with nature, where the idea of proximity to forests and waterways is commercially exploited as an addition to the notion of a “quiet and safe” life in the suburbs promoted by the “real estate privatopolis” defined by Hidalgo et al. (2008).

Viña del Mar exhibits high segregation of socially vulnerable populations in the upper hills, characterized by a high concentration of informal settlements, similar to Valparaíso. At the opposite extreme, its coastal strip is home to highly segregated upper socioeconomic groups (see Figure 2). This is linked to the intense processes of verticalization and financialization described by Valdebenito et al. (2020), which have concentrated along the entire coastline from Viña del Mar to Concón, where large-scale construction is driven by the touristification and capitalization of natural resources (Cáceres-Seguel, 2023; Valdebenito et al., 2020; Correa-Parra et al., 2025). In other words, Viña del Mar exemplifies a Latin American city where the most vulnerable must literally settle at the top of the hills in order to remain within the city.

Concón's expansion areas are composed of high-income groups residing along the coastal edge in high-rise developments and gated communities—projects designed to capitalize on views of the Pacific Ocean, as mentioned earlier regarding the development of the coastal area of Viña del Mar (Valdebenito et al., 2020). It is a polarized city, marked by high segregation among the wealthy in contrast to the low segregation observed in its historic urban areas near the city center (see Figure 2).

The commune of Quilpué exhibits high segregation of vulnerable groups in the Villa Olímpica and Belloto Norte areas, a process linked to the development of informal settlements and social housing complexes (see Figure 2). The areas of high residential segregation in the northern sector are associated with middle-income groups residing in newly developed real estate projects targeting this segment. This issue is specifically addressed by Vergara et al. (2023), who identify a serious problem with the price and availability of urban land in Valparaíso and Viña del Mar, which has resulted in a significant portion of new housing developments (for both vulnerable and middle-class households) having to be located in the municipalities of Quilpué and Villa Alemana, particularly in the outlying areas of those municipalities. This underscores the issues of low social and urban integration highlighted by Ruiz-Tagle and Romano (2019), as many of these neighborhoods—which consist of gated communities—suffer from limited access to public and private urban services, particularly public transportation, thereby reinforcing their functional dependence on Valparaíso.

In the case of the commune of Villa Alemana exhibits a significant difference between its northern and southern peripheries. The northern part displays residential expansion with low segregation (or greater social mix). This may be associated with the development of residential projects—or housing purchases—for middle-income groups (C2 or C3) in areas historically inhabited by low socioeconomic groups.

Meanwhile, the southern periphery is characterized by high and very high segregation associated with the development of social housing projects (Project DS49 and DS19, in Figure 4) and the construction of informal settlements (see Figure 2). Similar to Quilpué, it shows an “inner” city with low segregation, which contrasts with the high segregation of its southern expansion, linked to the possibility of urban expansion driven by the issue raised by Vergara (Hidalgo et al., 2023) regarding how State-subsidized housing complexes are being pushed out to the city's new outskirts.

### Location of social housing projects

An analysis of the residential integration or segregation conditions of social housing project residents shows that 70% of individuals with low social vulnerability live in areas of medium segregation, and 23.7% live in areas of high segregation. A highlight is the case of houses built under the DS 19 housing subsidy, a program aimed at social and territorial integration for middle socioeconomic groups. 36% of the beneficiaries of this subsidy live in areas of high or very high segregation, and 21% in areas of medium segregation (see Figure 4).

This means more than one-third of the households do not fulfill the subsidy's goal of achieving social integration. One possible explanation is the budgetary difficulty of accessing land within cities, which offer better social mix conditions. Suburban areas prove to be highly segregated spaces: on the one hand, vulnerable groups trying to stay in the city, and on the other, high-income groups seeking exclusivity or superior residential attributes.

The spatial distancing of social groups in the macro-territories of urban expansion outlines a periphery composed of highly differentiated residential-social fragments. This is relevant because Chile's Urban Policy includes among its objectives the reversal of current situations of urban social segregation (PNUD, 2018, p. 31). Evidence collected in the Metropolitan Area of Valparaíso shows that one-third of the projects aimed at this goal fail to meet the policy's objective. A direct factor is related to land prices, which hinders the construction of social housing in better-located areas. The city becomes a place for the enjoyment and exclusive habitation of high-income groups; land prices have become the city's new wall, condemning lower-income populations to life on urban margins. As Sabatini et al. (2001) emphasize, “the larger the size of homogeneous areas in poverty, the more severe the urban and social problems for their residents.” The peripheral expansion of subsidized housing redefines the social morphology of metropolitan areas, adding a category of trans-urbanite that, in order to access basic opportunities, must daily inhabit a city made up of fragmented and mutually distant territories (Cáceres, 2017).

## Discussion

The article's central argument is that subsidies—housing policy interventions—act as an active agent in the production of peripheries that replicate the segregating character of contemporary Chilean society. Following Ruiz-Tagle and Romano (2019), patterns of institutional segregation give rise to urban extension areas in Greater Valparaíso that can be understood as “institutional peripheries”: assemblages of State intervention mechanisms, intervention domains, resources, and agendas that produce these State spatialities at the edges of the Valparaíso metropolis. Over the last 30 years, the State has deepened this trend, generating segregated peripheries that it later seeks to change through the constant redefinition of its instruments, which non-etheless continue to exclude the availability of well-located land. Individual subsidies without tools to intervene in the land market only reproduce the current tendency toward segregated cities; they do not break the mechanism. This cycle repeats uninterruptedly because the goal of residential integration is included in policy documents but not in financing instruments capable of building the city beyond the dictates of the land market, thus externalizing the social costs of locations distant from services and opportunities. In this regard, Vergara and Riquelme (2024) argue that in cities marked by socioeconomic segregation (…), social-mix projects have less capacity to induce diverse sociabilities at the neighborhood scale.

The article highlights the relevance of the spatial factor and how the peripheral expansion areas of cities develop by producing socially homogeneous territories. As recognized in the literature, residential segregation in the AMV is expressed through socially homogeneous macro-territories and the concentration of vulnerable groups in the peripheries of the municipalities that make up the AMV (Sabatini et al., 2001). In dialogue with Fujita (2012), the AMV reflects a highly unequal society (Gini 0.46 in 2024) with a high degree of segregation of vulnerable groups, in a trend that appears to be intensifying as Chilean cities continue to expand. Moreover, the study shares the findings of a peripheral location of social housing. In this regard, authors have pointed out (Rasse Figueroa et al., 2021) that the objective of reducing the housing deficit has gone hand in hand with the peripheral siting of housing in the AMV.

The suburban expansion zones of the Valparaíso Metropolitan Area do not develop with social mixing, but rather with higher levels of segregation than the rest of the city. It shows that, far from correcting historical inequalities, recent urban growth (1992–2017) is accentuating and intensifying social exclusion in the metropolitan area. This process involves the convergence of both the peripheral location of vulnerable groups in social housing projects and informal settlements—groups that can only access the city's edges. The suburban expansion areas built between 1992 and 2017, driven by both public and private action, have acted as a social centrifuge, distancing social groups from each other across a vast macro-territory at the city's edge. This segregation generates diverse forms of exclusion, such as precarious public transportation that complicates daily commutes to employment centers located in Viña del Mar and Valparaíso and restricts movement on holidays or during the night. Drawing on Toscana (2017), the residential peripheries of the MAV reproduce “durable structures” of social segregation that generate a differentiated space that will persist for decades.

## Conclusion

This geography of residential segregation in the MAV should not be viewed disconnected from the spatialities of the State, a State that, through gaps and decisions, deploys a social-spatial hierarchy concentrating vulnerable groups in specific areas. A revealing fact is that one-third of the housing built for a social integration program is located in areas with high social segregation. This demonstrates the need for instruments that allow confronting the segregating tendencies derived from land market dynamics. The instruments designed to promote social mixing are, in fact, exacerbating segregation in new growth areas. The research demonstrates that subsidies intended for projects with the explicit objective of “social mixing” end up located precisely in highly segregated areas. This reveals a critical disconnection between the intention of public policy (inclusion and integration) and its effective spatial result (greater segregation). Following Jessop (1990), the mere existence of the State—as a distinctive entity of social relations—does not ensure a coherent and coordinated framework for State action; rather, it emerges as an actor configured by strategies of action in dispute.

The data warns of the need for new instruments and land availability to intensify the development of these projects in more central areas with a greater social mix. A relevant aspect to be included in future research concerns understanding how nationality or origin operates in the configuration of segregated areas in the AMV—an issue considered significant by researchers (Ruiz-Tagle and López, 2014). It is relevant to promote the development of real estate projects for middle-income groups in vacant spaces within sectors that currently house lower socioeconomic groups—plans that combine DS projects with the improvement of neighborhood infrastructure and equipment. The peripheries of the MAV are returned to us as mirrors of a highly segregated society. New urban planning instruments are urgently needed because the peripheries will define a large part of the sustainability and social cohesion of Chilean society. This finding provides crucial empirical evidence for the debate on the effectiveness of urban and social tools implemented in the region, offering valuable lessons that transcend Chile and are applicable to other Latin American metropolises that use similar subsidy models.

Although this article addressed the segregation of vulnerable groups in the peripheries, it is important to note that the segregation of vulnerable groups—triggered by the action or inaction of the State—is simultaneously reproduced in central neighborhoods as well as at the edges of the metropolis. At times, urban centers become elitized while the peripheries accommodate vulnerable groups; in other cases, centers become precarious while elites inhabit gated projects in the peripheries. Therefore, combating segregation requires effective instruments and political decisions on the part of States. First, it involves understanding that social housing policies cannot be developed disconnected from effective mechanisms such as control of public land, metropolitan planning oriented by public transport, recycling of central urban areas, and the creation of metropolitan subcenters that decentralize employment and services. Second, it involves generating incentives for the private sector to invest in real estate projects that include percentages of public housing. We will not build inclusive cities without including the private sector in the common city project. These geographies of social inequality impact planetary-scale objectives such as the fight against inequality, sustainable mobility, access to housing, and policies in response to migratory waves, turning the peripheries into strategic spaces for the urban future of the planet.

## Data Availability

The original contributions presented in the study are included in the article/supplementary material, further inquiries can be directed to the corresponding author.

## References

[B1] Cáceres C. (2017). Vivienda social periurbana en Santiago de Chile: la exclusión a escala regional del trasurbanita de Santiago de Chile. Econ. Soc. Territ. 53, 171–198 doi: 10.22136/est002017664

[B2] Cáceres C. y Ahumada G. (2020). Acceso a equipamiento urbano y calidad de vida. Quilpué y Villa Alemana, Chile. Bitácora Urbano Territorial 30, 263–275. doi: 10.15446/bitacora.v30n3.86844

[B3] Cáceres-Seguel C. (2023). Valparaíso: touristification and displacement in a UNESCO city. J. Urban Aff. 48, 1–13. doi: 10.1080/07352166.2023.2203400

[B4] Correa-Parra J. Vergara-Perucich F. Aguirre-Nuñez C. Ulloa-León F. I. (2025). The 15-min city and real estate capital: contributions from Chile's major cities. Town Plan. Rev. 96, 671–696. doi: 10.3828/tpr.2025.8

[B5] Correa-Parra J. Vergara-Perucich F. Rodríguez-Valladares N. Aguirre-Nuñez C. Hidalgo-Dattwyler R. (2023). Desafíos de integración urbana en chile: segregación residencial y el rol del capital humano avanzado bajo influencias neoliberales. Rev. Urban. 49, 115–137. doi: 10.5354/0717-5051.2023.71506

[B6] Fernández-de-Córdova G. Moschella P. Fernández-Maldonado A. M. (2021). “Changes in Spatial Inequality and Residential Segregation in Metropolitan Lima,” in Urban Socio-Economic Segregation and Income Inequality. The Urban Book Series. eds. M. van Ham, T. Tammaru, R. Ubarevičiene, and H. Janssen (Cham: Springer), 311–332.

[B7] Fuentes L. y Pezoa M. (2017). Crecimiento urbano reciente del gran Valparaí*so. ¿hacia una reconfiguración com-fusa? Revista* 180, (40). Available online at: https://revista180.udp.cl/index.php/revista180/article/view/328 (Accessed September 15, 2025).

[B8] Fujita K. (2012). “Conclusion: residential segregation and urban theory,” in Residential Segregation in Comparative Perspective: Making Sense of Contextual Diversity, eds. T. Maloutas and K. Fujita (Surrey: Ashgate Publishing), 285–322.

[B9] Fuster-Farfán X. Ruiz I. Henry L. (2023). Las periferias de la periferia: producción de ciudad y política habitacional en Chile. Territorios 49, 1–28.doi: 10.12804/revistas.urosario.edu.co/territorios/a.12404

[B10] Garreton M. Basauri A. Valenzuela L. (2020). Exploring the correlation between city size and residential segregation: comparing Chilean cities with spatially unbiased indexes. Environ. Urban. 32, 569–588. doi: 10.1177/0956247820918983

[B11] Habitat World Map UN (2019). Available online at: https://habitat-worldmap.org/en/key-words/social-productionof-habitat/

[B12] Hidalgo A. Borsdorf A. Zunino H. Alvarez L. (2008). Tipologías de expansión metropolitana en Santiago de Chile: precariópolis estatal y privatópolis inmobiliaria. Script Anova XII:270.

[B13] Hidalgo R. Vergara C. Correa J. Alvarado V. Robles M. Rodriguez N. eds. (2023). Vivir en la punta del cerro: vivienda subsidiada, segregación y producción de naturaleza en el área metropolitana del Gran Valparaíso en Ferrara. Natureza e Metabolismo Urbano: reestruturação do espaço no Brasil e no Chile 1, 329–361.

[B14] Instituto Nacional de Estadísticas (2017). Censo de población y vivienda 2017. Santiago, Instituto Nacional de Estadí*sticas*. Available online at: https://redatam-ine.ine.cl/

[B15] Jessop B. (1990). State Theory: Putting the Capitalist State in Its Place. Polity Press.

[B16] Ministerio de Vivienda y Urbanismo (MINVU) (2014). Hacia una nueva política urbana para Chile. Política Nacional de Desarrollo Urbano. Santiago: Ministerio de Vivienda y Urbanismo. Available online at: http://cndu.gob.cl/wp-content/uploads/2014/10/L4-Politica-Nacional-Urbana.pdf

[B17] Ministerio de Vivienda y Urbanismo (MINVU), (2020). Mapa del continuo de construcciones urbanas de ciudades chilenas 1993-2020. Available online at: https://ide.minvu.cl/maps/81f33821d6c84a378b8e754d9f2a6a45 (Accessed September 15, 2025).

[B18] Observatorio de Ciudades UC (OCUC), (2023). Metodologí*a Índice Socio Material Territorial, ISMT*. Available online at: https://github.com/ObervatorioCiudadesUC/ISMT (Accessed September 15, 2025).

[B19] Pino A. Ojeda L. (2013). Ciudad y hábitat informal: las tomas de terreno y la autoconstrucción en las quebradas de Valparaíso. Revista INVI 28, 109–140. doi: 10.4067/S0718-83582013000200004

[B20] PNUD, (2018). Desigualdad regional en Chile. Ingresos, salud y educación en perspectiva territorial. Santiago de Chile, Programa de las Naciones Unidas para el Desarrollo. Available online at: https://www.estudiospnud.cl/informes-desarrollo/desigualdad-regional-en-chile-ingresos-salud-y-educacion-en-perspectiva-territorial/ (Accessed September 15, 2025).

[B21] Rasse Figueroa A. Sarella Robles M. Sabatini Downey F. Cáceres Quiero G. y Trebilcock M. P. (2021). Desde la segregación a la exclusión residencial. ¿Dónde están los nuevos hogares pobres (2000–2017) de la ciudad de Santiago, Chile? Revista de Urbanismo 44, 39–59. doi: 10.5354/0717-5051.2021.55948

[B22] Rojo-Mendoza F. Alvarado Peterson V. (2023). Dinámicas de estratificación socioespacial en ciudades del norte y sur de Chile. Rev. Urbanismo 85–109. doi: 10.5354/0717-5051.2023.68011

[B23] Ruiz-Tagle J. López E. (2014). El estudio de la segregación residencial en Santiago de Chile: revisión crítica de algunos problemas metodológicos y conceptuales. EURE 40, 25–48. doi: 10.4067/S0250-71612014000100002

[B24] Ruiz-Tagle J. Romano S. (2019). Mezcla social e integración urbana: aproximaciones teóricas y discusión del caso chileno. INVI 34, 45–69. doi: 10.4067/S0718-83582019000100045

[B25] Sabatini F. Brain I. (2008). La segregación, los guetos y la integración social urbana: Mitos y claves. Eure 34, 5–26. doi: 10.4067/S0250-71612008000300001

[B26] Sabatini F. Cáceres G. Cerda J. (2001). Segregación residencial en las principales ciudades chilenas: tendencias de las tres últimas décadas y posibles cursos de acción. EURE 27, 21–42. doi: 10.4067/S0250-71612001008200002

[B27] Sabatini F. Rasse A. Cáceres G. Robles M. S. Trebilcock M. (2017). Promotores inmobiliarios, gentrificación y segregación residencial en Santiago de Chile. Rev. Mex. Sociol. 79, 229–260. doi: 10.22201/iis.01882503p.2017.2.57662

[B28] Sassen S. (1991). The Global City: New York, London, Tokyo. Princeton, NJ: Princeton University Press.

[B29] Tammaru T. Knapp D. Silm S. van Ham M. Witlox F. (2021). Spatial underpinnings of social inequalities: a vicious circles of segregation approach. Soc. Incl. 9, 65–76. doi: 10.17645/si.v9i2.4345

[B30] Theil H. Finizza A. J. (1971). A note on the measurement of racial integration of schools by means of informational concepts. J. Math. Sociol. 1, 187–193. doi: 10.1080/0022250X.1971.9989795

[B31] Toscana A. (2017). En busca de la justicia espacial. Polít. Cult. 48, 207–211. doi: 10.24275/LRVN2657

[B32] Valdebenito C. Álvarez L. Hidalgo R. y Vergara C. (2020). Transformaciones sociodemográficas y diferenciación social del espacio residencial en el área metropolitana de Valparaíso, Chile (1992–2017). Investig. Geogr. 74, 271–290. doi: 10.14198/INGEO2020.VVAAHDVC

[B33] Van Ham M. Tammaru T. Ubareviciene R. Janssen H. (eds.). (2021). Urban Socio-economic Segregation and Income Inequality: A Global Perspective. The Urban Book Series. Cham: Springer. doi: 10.1007/978-3-030-64569-4

[B34] Vergara C. Hidalgo R. Correa J. Alvarado V. Sarella M. Rodriguez N. (2023). “Vivir en la punta del cerro: vivienda subsidiada, segregación y producción de naturaleza en el área metropolitana del Gran Valparaíso,” in book: Natureza e metabolismo urbano: reestruturação do espaço no Brasil e no Chile. (Rio de Janeiro: Letra Capital), 329–361.

[B35] Vergara L. Riquelme A. (2024). Neo-liberalized housing policy and urban accessibility: the relevance of perception in intermediate cities. the case of temuco, chile. J. Hous. Built Environ. 39, 1–20 doi: 10.1007/s10901-023-10082-6

